# Novel Molecular Subtyping Predicts Locoregional Recurrence in Triple-negative Breast Cancer

**DOI:** 10.1016/j.adro.2025.101909

**Published:** 2025-10-02

**Authors:** Shali Shao, Wei Shi, Li Zhang, Qixian Zhang, Jin Meng, Zhaozhi Yang, Miao Mo, Zhen Zhang, Xiaomao Guo, Xiaoli Yu

**Affiliations:** a Department of Radiation Oncology, Women's Hospital, School of Medicine, Zhejiang University, Hangzhou, Zhejiang, China; b Zhejiang Provincial Key Laboratory of Precision Diagnosis and Therapy for Major Gynecological Diseases, Women's Hospital, Zhejiang University School of Medicine, Hangzhou, People's Republic of China; c Zhejiang Provincial Clinical Research Center for Obstetrics and Gynecology, Hangzhou, Zhejiang, China; d Department of Radiation Oncology, Fudan University Shanghai Cancer Center, Shanghai, China; e Department of Oncology, Shanghai Medical College, Fudan University, Shanghai, China; f Shanghai Clinical Research Center for Radiation Oncology, Shanghai, China; g Shanghai Key Laboratory of Radiation Oncology, Shanghai, China; h Department of Cancer Prevention and Clinical Statistics Center, Fudan University Shanghai Cancer Center, Shanghai, China

## Abstract

**Purpose:**

Immunohistochemistry (IHC)-based molecular subtyping provides a comprehensive profile of triple-negative breast cancer (TNBC). We aimed to elucidate patterns of locoregional recurrence (LRR) in different subtypes of TNBC.

**Methods and Materials:**

In this study, 352 patients with breast cancer treated with mastectomy and postmastectomy radiation therapy from November 2019 to March 2022 were retrospectively analyzed. Based on IHC, these patients were classified into the basal-like immune-suppressed (BLIS) and non-BLIS groups. We compared LRR as the first event, disease-free survival, and overall survival (OS) between the 2 groups. Univariate and multivariate analysis was performed.

**Results:**

The median follow-up time was 44.6 months. Twenty-six LRR (including 9 isolated LRR) and 70 distant metastases (DMs) were observed. The cumulative incidence of LRR was significantly different between the 2 groups, with an LRR rate of 5.8% in the non-BLIS group and 14.9% in the BLIS group (*P* = .005). The difference in OS was also significant (91.9% vs 83.0%, *P* = .01). However, there was no significant difference in disease-free survival between the non-BLIS and BLIS groups (80.2% vs 74.5%, *P* = .35). Multivariate analysis demonstrated that IHC-based molecular subtyping was an independently prognostic factor for LRR and OS (*P* = .03; *P* = .03).

**Conclusions:**

This study demonstrated that the BLIS subtype appears to be at a higher risk of developing LRR, and IHC-based molecular subtyping might be used as prognostic biomarkers to guide postmastectomy radiation therapy in patients with TNBC. New strategies that improve locoregional control rates in patients with BLIS are warranted.

## Introduction

Triple-negative breast cancer (TNBC) is characterized by a lack of expression of estrogen receptor (ER), progesterone receptor (PR), and human epidermal growth factor receptor 2 (HER2). TNBC accounts for approximately 10% to 15% of all breast cancers, which is notably lower than other subtypes.^1^^,^^2^ Nevertheless, TNBC carries a notably poor prognosis, marked by a high risk of early recurrence and a greater propensity for visceral metastases.^3^^,^^4^

The TNBC cohort exhibits a markedly unfavorable prognosis, manifesting higher rates of local recurrence (LR) at 7.3% and regional recurrence (RR) at 3.3% compared with the luminal cohort.^5^ The locoregional recurrence (LRR) pattern among patients with TNBC is markedly distinguished from that of other subtypes. It reaches its peak about one year after surgery and gradually decreases thereafter.^6^ Several randomized clinical trials and the meta-analysis conducted by the Early Breast Cancer Trialists’ Collaborative Group have shown that postmastectomy radiation therapy (PMRT) reduces LRR and enhances survival in patients with high-risk breast cancer.^7^^,^^8^^.^ Although a biological subtype has been demonstrated to be a prognostic factor for LRR in PMRT, and there is evidence of differences in radiosensitivity among breast cancer subtypes, there are no guidelines for adjusting radiation volume.

Recently, increasing evidence has revealed the highly heterogeneous nature of TNBC, which impacts the treatment strategies significantly. In 2011, Lehmann et al^9^ categorized TNBC into 6 molecular subtypes using gene expression cluster analysis. Burstein et al^10^ identified 4 distinct subtypes of TNBC through RNA and DNA profiling. Based on multiomic profiling of 465 Chinese patients with TNBC, Jiang et al^11^ classified TNBC cases into 4 messenger RNA subtypes: luminal androgen receptor (LAR), immunomodulatory (IM), basal-like immune-suppressed (BLIS), and mesenchymal-like (MES). The identification of these molecular subtypes is crucial for understanding the heterogeneity of TNBC and guiding individualized treatment decisions. However, the wide application of TNBC molecular subtypes in clinical practice is hindered by high costs and technological complexity. To address this issue, an immunohistochemistry (IHC)-based classifier was proposed using 4 markers (androgen receptor [AR], CD8, forkhead box transcription factor C1 [FOXC1], and doublecortin-like kinase 1[DCLK1]). This greatly improves clinical feasibility.^12^^,^^13^

A better understanding of the phenotypic characteristics and recurrence patterns of TNBC is important for personalized treatments. However, there are few studies on the feasibility of molecular typing as a prognostic factor for PMRT in patients with TNBC. Previous research has noted that BLIS subtypes exhibit the worst prognosis,^14^ yet whether BLIS has worse locoregional control after PMRT remains unknown. In this study, we retrospectively analyzed our institutional database of patients with TNBC and classified them into BLIS groups and non-BLIS molecular subtypes using an IHC-based approach. We aimed to investigate the patterns of LRR and predictors of disease-free survival (DFS) and overall survival (OS).

## Methods and Materials

### Study design and participants

From November 2019 to March 2022, data from 352 American Joint Committee on Cancer 7th edition stages II to III patients with breast cancer treated with mastectomy and PMRT with or without neoadjuvant chemotherapy (NAC) in Fudan University Shanghai Cancer Center were retrospectively analyzed. Data in this article were approved by the Ethical Committee in the cancer center. Clinicopathologic details (laterality, surgery date, pathologic stage, grade, and lymphovascular invasion), treatment information (chemotherapy regimens, surgical type, adjuvant therapy, and PMRT dose and fractionation) were collected. The status of ER, PR, and HER2 was confirmed using IHC analysis. According to the American Society of Clinical Oncology/College of American Pathologists guideline,^15^ ER/PR negativity in IHC is defined as less than 1% positively stained cells. HER2 negativity is defined as the stain score of HER2 under 1 or the absence of HER2 gene amplification using fluorescent in situ hybridization. Complete molecular subtypes-specific biomarker information (AR, CD8, FOXC1, and DCLK1) was confirmed using IHC. Patients with a preliminary diagnosis of DMs, occult breast carcinoma, a previous history of contralateral breast cancer or other malignancies, and previous breast radiation therapy (RT) were excluded.

### IHC-based molecular subtyping

**Figure 1:**
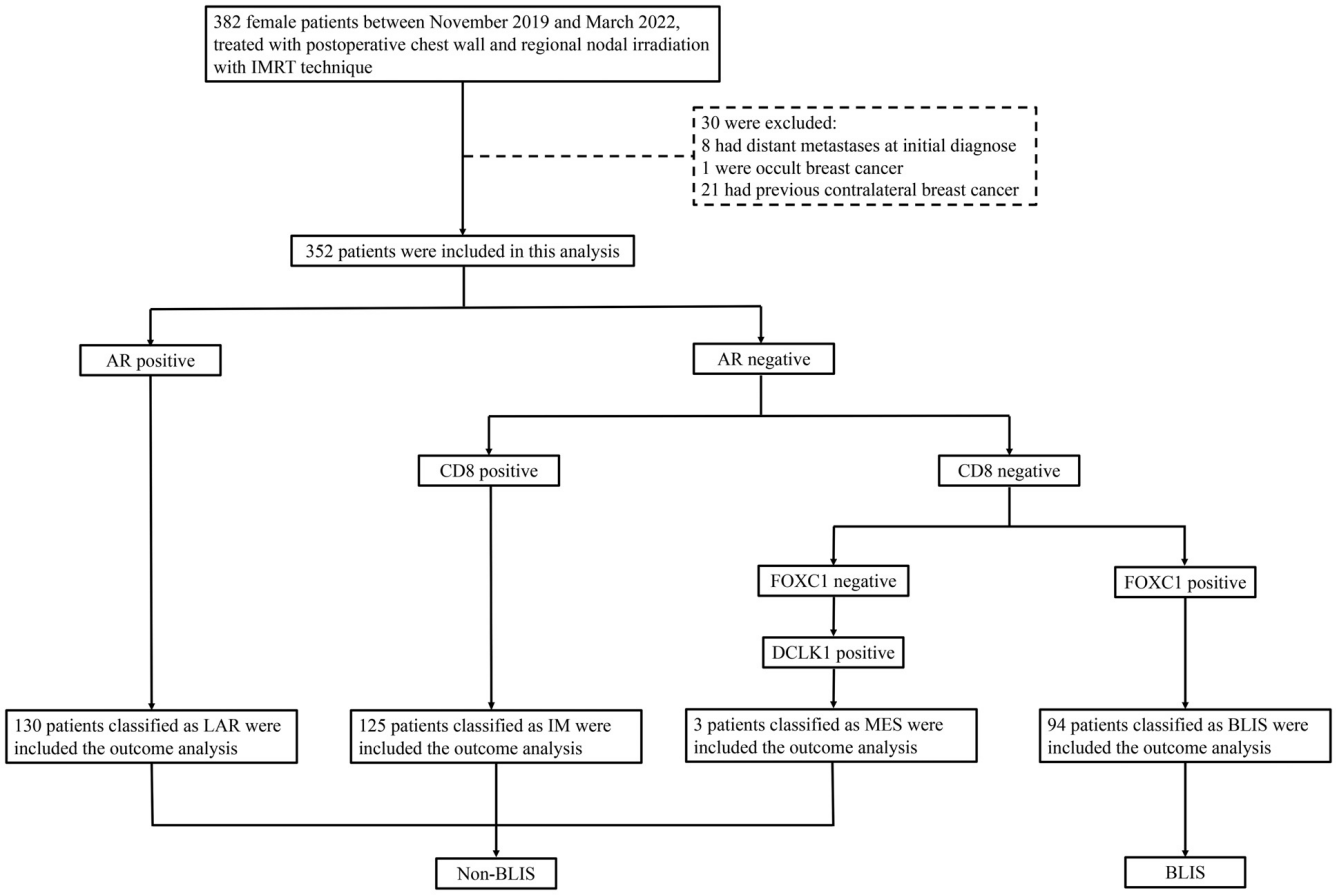
CONSORT (Consolidated Standards of Reporting Trials) flow diagram. *Abbreviations*: BLIS = basal-like immune-suppressed; LAR = luminal androgen receptor; IM = immunomodulatory; IMRT = intensity modulated radiation therapy; MES = mesenchymal-like.

Expression of AR, CD8, FOXC1, and DCLK1 in at least 10% of tumor cells using IHC was defined as AR, CD8, FOXC1, and DCLK1 positive. Tumors were classified into different subtypes based on the IHC classifier developed by the team of Fudan University Shanghai Cancer Center. AR+ was classified as IHC-based LAR; AR− and CD8+ were classified as IHC-based IM; AR−, CD8−, and FOXC1+ were classified as IHC-based BLIS; and AR−, CD8−, FOXC1−, and DCLK1− were classified as IHC-based MES. The patients were divided into LAR, IM, MES, and BLIS groups according to the IHC classifier, and then LAR, IM, and MES were combined into the non-BLIS group (Fig. 1).

### Surgery and systemic treatment

Surgery included mastectomy and axillary lymph node dissection. All patients underwent mastectomy, and 94.3% had axillary lymph node dissection. All the patients in this study received systemic therapy (NAC, adjuvant chemotherapy, or both). Nearly half of the patients (41.5%) received NAC. The most common regimens in the NAC setting were taxane-based regimens, such as paclitaxel combined with carboplatin and epirubicin plus cyclophosphamide, followed by docetaxel. In the overall population, a majority of patients (92.0%) received adjuvant chemotherapy. The most commonly used regimens were anthracycline-based regimens. After NAC, surgery, and adjuvant chemotherapy, a small number of patients (15.4%) received consolidation therapy, with capecitabine being the most common one.

### Adjuvant RT

The patient was placed in the supine position, fixed with a breast tilt board (Med-Tech 350), with both arms raised and placed on the armrests. The head was centrally aligned or deviated to the contralateral side, with the lower jaw slightly raised to avoid neck skin folds. Computed tomography (CT) scan simulation was performed using an AcQsim CT simulator (Philips Medical Systems) with a slice thickness of 5 mm. Before CT scanning, dots are used to mark clinically visible surgical scars. The CT scan range was from the basis cranii to the diaphragm.

The RT target volume commonly included the ipsilateral chest wall, supraclavicular, and undissected axillary with or without the internal mammary node (IMN) region. In general, the delineation of clinical target volume (CTV) was based on Radiation Therapy Oncology Group and European Society for Radiotherapy and Oncology (ESTRO) consensus guidelines.^16^^,^^17^ However, for some controversial areas, modifications have been made at our cancer center to establish institutional guidelines. The borders of the chest wall CTV were the same as the definitions of the ESTRO consensus guidelines, except for the ventral border, which was the surface of skin, because of the thin chest wall in Chinese women. For patients with T4a/T4c disease, the major pectoral muscles and ribs should be included. The surgical scar should be routinely included in the chest wall CTV. The chest wall planning target volume (PTV) was expanded 5 mm from the chest wall CTV. An additional chest wall PTV for evaluation is generated by limiting the ventral border of the chest wall PTV at the skin surface for planning evaluation. Regional nodal irradiation (RNI) was based on lymph nodal status and mainly includes supraclavicular and undissected axillary, with or without the IMN region. Patients with pathologically negative lymph nodes may be omitted from RNI. The delineation of supraclavicular CTV included medial supraclavicular CTV or entire supraclavicular CTV, which was determined by individual radiation oncologists’ preference. The medial supraclavicular CTV’ dorsal border was the anterior aspect of the scalene muscle, whereas the entire supraclavicular CTV’ dorsal border was the trapezius muscle. Indications for IMN irradiation were as follows: (1) pN2-3; (2) lymph node+ patients with centrally or medially located tumors; (3) IMN metastasis. The borders of IMN CTV were the same as the definitions of the ESTRO consensus guidelines, including the internal mammary vessels in the first 3 intercostal spaces. Undissected axillary CTV consisted of the undissected portions of the axilla, typically including level 3, interpectoral nodes, and part of axillary level 2. The PTVs were generated by expanding 0.5 cm from the CTVs.

The RT regimens were conventional fractionation (50 Gy/25 Fx) and hypofractionated RT (42.56 Gy/16 Fx). For lesions with a high suspicion of residual disease based on imaging, a local and regional boost to 60 to 66 Gy may be administered. A 3 mm bolus was placed on the chest wall to ensure an adequate skin dose. Intensity modulated RT planning was used. The planning optimization was guided with dose-volume constraints as follows: 95% of the PTV received at least 95% of the prescription dose (D95 > 95%). For right-sided breast cancer, the mean dose of heart (Dmean) < 2 Gy; for left-sided breast cancer, the heart Dmean < 8 Gy; the ipsilateral lung V5 Gy < 70%, and V20 Gy < 35%; the contralateral lung V5 Gy < 15%; the maximum dose of spinal cord (Dmax) < 45 Gy; the trachea Dmean < 25 Gy; the ipsilateral humeral head Dmean < 25 Gy; the thyroid glands Dmean < 25 Gy; and the ipsilateral brachial plexus Dmax < 60 Gy.

### Follow-up and outcomes

Patients were followed every 3 months for the first 2 years postoperatively, twice annually in years 3 to 5, and annually thereafter. Follow-up mainly included physical examination, laboratory tests, and imaging. LRR was defined as any first breast cancer recurrence occurring in the ipsilateral chest wall or regional lymph nodes, with or without concurrent distant metastasis, including ipsilateral axillary, supraclavicular area, or IMN. DFS was defined as the interval from the date of breast surgery to LRR, DMs, or death from any cause. OS was defined as the time from surgery to death from any cause. Pathologic complete response (pCR) was defined as the absence of residual invasive disease in the breast and axilla.

### Statistical analysis

Basic clinicopathologic information was presented. Frequencies and percentages were used to describe categorical variables, whereas continuous variables were reported as the mean ± standard deviation. The comparison between groups was performed using χ^2^ or Fisher’s exact tests for categorical variables and the Mann-Whitney test for continuous data. The cumulative incidence function was applied to estimate LRR rates, with death as a competing risk, using Gray's test for comparisons. The survival probabilities of DFS and OS were analyzed using Kaplan-Meier curves and compared using the log-rank test. Age, tumor size, lymph node involvement, grade, lymphovascular invasion (LVI), and IHC-based molecular subtyping were included in univariate and multivariate analysis. A competing risk regression model was used for the univariate and multivariate analysis of LRR, and the Cox regression model was used for DFS and OS analysis. Statistical analyses were performed using SPSS version 24.0 (SPSS) and R version 4.1.3. A 2-sided *P* value < .05 indicates statistical significance.

## Results

### Patients and treatment characteristics

A total of 352 patients with TNBC were retrospectively analyzed. According to the IHC results, 130 (36.9%), 125 (35.5%), 3 (0.8%), and 94 (26.7%) patients were classified into LAR, IM, MES, and BLIS subtypes, respectively. A total of 258 patients belonged to the non-BLIS group and 94 to the BLIS group. Patients and treatment characteristics are presented in Table 1. The median age was 50 years (range, 24-82 years). Most of them were T0-2 (91.8%), N0-2 (86.6%), and had positive LVI (61.9%). Except that the patients in the BLIS group were younger (mean age 46 years), other baseline characteristics were balanced between the 2 groups.

**Table 1:** Baseline clinicopathologic and treatment characteristics of patients

Characteristics		Non-BLIS (n = 258) n (%)	BLIS (n = 94) n (%)	Total (n = 352) n (%)	*P* value
Age, y	Mean ± SD	51.76 ± 11.15	46.00 ± 11.95	50.22 ± 11.63	< .001
Laterality	Left	132 (51.2)	48 (51.1)	180 (51.1)	.99
	Right	126 (48.8)	46 (48.9)	172 (48.9)	
Pathologic category, T	T0-2	241 (93.4)	82 (87.2)	323 (91.8)	.06
	T3-4	17 (6.6)	12 (12.8)	29 (8.2)	
Pathologic category, N	N0-2	222 (86.0)	83 (88.3)	305 (86.6)	.58
	N3	36 (14.0)	11 (11.7)	47 (13.4)	
Grade	II	47 (18.2)	15 (16.0)	62 (17.6)	.84
	III	162 (62.8)	62 (66.0)	224 (63.6)	
	Unknown	49 (19.0)	17 (18.0)	66 (18.8)	
Lymphvascular invasion	Yes	157 (60.9)	61 (64.9)	218 (61.9)	.78
	No	72 (27.9)	23 (24.5)	95 (27.0)	
	Unknown	29 (11.2)	10 (10.6)	39 (11.1)	
Systemic treatment	Neoadjuvant chemotherapy	22 (8.5)	6 (6.4)	28 (8.0)	.05
	Adjuvant chemotherapy	159 (61.6)	47 (50.0)	206 (58.5)	
	Both	77 (29.8)	41 (43.6)	118 (33.5)	
Fractionation regimens	HF	49 (19.0)	12 (12.8)	61 (17.3)	.17
	CF	209 (81.0)	82 (87.2)	291 (82.7)	
IMN irradiation	Yes	215 (83.3)	80 (85.1)	295 (83.8)	.69
	No	43 (16.7)	14 (14.9)	57 (16.2)	

*Abbreviations:* BLIS = basal-like immune-suppressed; CF = conventional fractionation; HF = hypofractionated fractionation; IMN = internal mammary node; SD = standard deviation.

### Adjuvant RT

All patients received PMRT. The most common PMRT regimen was 50 Gy in 25 fractions (82.6%), whereas 61 patients received 42.56 Gy in 16 fractions (17.3%). A total of 349 patients received RNI (99.1%), of whom 295 received IMN irradiation (84.5%). RNI was omitted in 3 patients because of negative lymph node pathology. Boost was delivered to the postmastectomy chest wall, supraclavicular area, or IMN in 24 (6.8%), 27 (7.7%), and 13 (3.7%) patients.

### The BLIS subtype has a higher LRR rate than the non-BLIS

**Figure 2:**
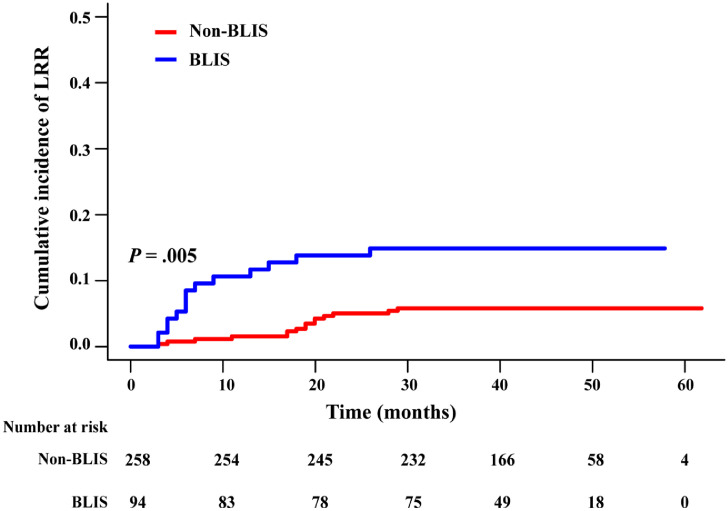
The cumulative incidence of local and regional recurrence of the nonbasal-like immune-suppressed (BLIS) group and BLIS group. Fine-Gray test was used. *Abbreviation:* LRR = locoregional recurrence.

The median follow-up time was 44.6 months (range, 8.8-67.3 months). The LRR rate for the entire cohort was 8.24%. The cumulative incidence of LRR between the 2 groups was significantly different, with a rate of 5.8% in the non-BLIS group and 14.9% in the BLIS group (*P* = .005) (Fig. 2). Table 2 shows the patterns of LRR. Of the 26 patients with LRR as the first event, 9 had isolated LRR and 17 were diagnosed with LRR with concurrent DMs: 11 synchronously (within 3 months) and 6 subsequently. Among the 26 patients with LRR, 15 had local recurrence, and 15 had RR (4 simultaneously). The most common area of recurrence was the supraclavicular region. The majority of recurrences (80.8%, 21/26) occurred within the RT field (in-field), and no isolated out-of-field relapse was observed. The results of univariate and multivariate analyses of LRR for the whole cohort are shown in Table 3. In multivariate analysis, independent factors predicting higher LRR were BLIS subtype (hazard ratio [HR], 2.52; 95% confidence interval [CI], 1.10-5.78, *P* = .03). LVI, grade, N stage, and T stage were not independent prognostic factors for LRR.

**Table 2:** Patterns of LRR and DM

Site	Non-BLIS (n = 258) n (%)	BLIS (n = 94) n (%)
Any breast cancer recurrence	56 (21.7)	24 (25.5)
Local-regional recurrence (ipsilateral)	12 (4.7)	14 (14.9)
Isolated	7 (2.7)	2 (2.1)
With simultaneous distant metastases	5 (1.9)	12 (12.8)
Local recurrence (ipsilateral)	7 (2.7)	8 (8.5)
Regional recurrence (ipsilateral)	9 (3.5)	6 (6.4)
Axillary	5 (1.9)	2 (2.1)
Supraclavicular	7 (2.7)	4 (4.3)
Internal mammary	1 (0.4)	0
Distant metastases	49 (19.0)	21 (22.3)
Death (any cause)	21 (8.1)	16 (17.0)
Breast cancer causes	21 (8.1)	16 (17.0)
Nonbreast cancer causes	0	0
Second cancer	10 (3.9)	3 (3.2)
Contralateral breast cancer	0	0
Other primary malignancies	10 (3.9)	3 (3.2)

*Abbreviations:* BLIS = basal-like immune-suppressed; DM = distant metastasis; LRR = locoregional recurrence.

**Table 3:** Univariate and multivariate competing risk regression model analysis of LRR

Variables		UVA (LRR) HR 95% CI	*P* value	MVA (LRR) HR 95% CI	*P* value
Age, y		0.97 (0.94-1.00)	.02	0.98 (0.94-1.01)	.20
Subtype	Non-BLIS	1	1		
	BLIS	2.77 (1.34-5.72)	<.01	2.52 (1.10-5.78)	.03
Lymphvascular invasion	Positive	1	1		
	Negative	0.44 (0.15-1.28)	.13	0.41 (0.12-1.45)	.17
	Unknown	1.41 (0.53-3.72)	.49	1.19 (0.41-3.48)	.75
Pathologic category, T	0-2	1	1		
	3-4	2.40 (0.93-6.17)	.07	2.21 (0.82-6.00)	.12
Pathologic category, N	1-2	1	1		
	3	3.11 (1.43-6.79)	<.01	2.65 (1.02-6.91)	.05
Grade	II	1	1		
	III	0.68 (0.27-1.75)	.43	0.63 (0.22-1.85)	.40
	Unknown	1.26 (0.44-3.59)	.67	1.36 (0.40-4.60)	.62

*Abbreviations*: BLIS = basal-like immune-suppressed; CI = confidence interval; HR = hazard ratio; LRR = locoregional recurrence; MVA = multivariate analysis; UVA = univariate analysis.

### BLIS subtype portends unfavorable prognosis than the non-BLIS subtype

**Figure 3:**
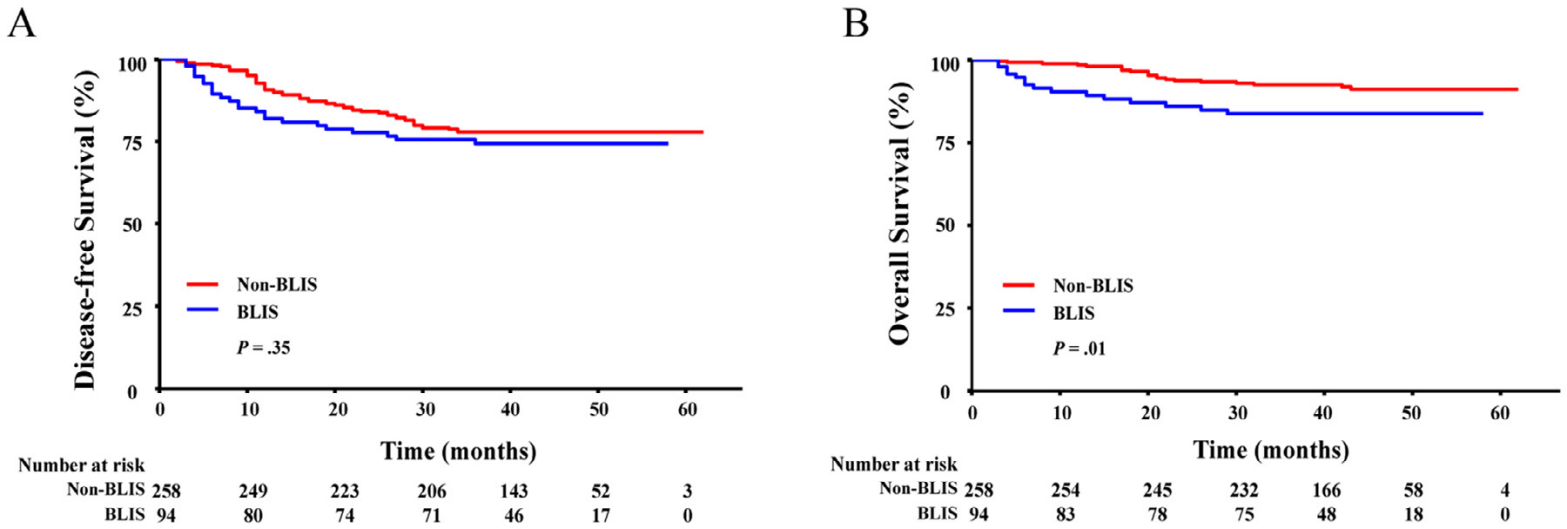
Disease-free survival (A) and overall survival (B) of the non-basal-like immune-suppressed (BLIS) group and BLIS group.

In the entire cohort, 80 of 352 (22.7%) patients experienced recurrent breast cancer. The most common sites of metastases were the liver, brain, and lung. The DFS rate for the entire cohort was 77.3%, and the OS rate was 89.5%. The 4-year DFS rates were 80.2% and 74.5% (*P =* .35) for non-BLIS and BLIS subtypes, respectively. The 4-year OS rates were 91.9% and 83.0% (*P* = .01), respectively (Fig. 3). In univariable analysis, subtype (HR, 2.25; 95% CI 1.18-4.32, *P* = .01), pT3-4 (HR, 2.29; 95% CI, 1.24-4.24, *P* < .01), and pN3 (HR, 4.01; 95% CI, 2.51-6.40, *P* < .001) were associated with worse OS. Multivariate analysis showed that subtype, pT3-4, and pN3 (HR, 2.16; 95% CI, 1.09-4.28, *P* = .03; HR, 2.17, 95% CI, 1.12-4.23, *P* = .02; and HR, 3.56; 95% CI, 2.16-5.88, *P* < .001) were also independently associated with worse OS (Table S1). Univariate (HR, 2.29; 95% CI, 1.24-4.24, *P* < .01; HR, 4.01; 95% CI, 2.51-6.40, *P* < .001) and multivariate (HR, 2.17; 95% CI, 1.12-4.23, *P* = .02; HR, 3.56; 95% CI, 2.16-5.88, *P* < .001) analyses showed that pT3-4 and N3 were independent factors for DFS (Table S2).

## Discussion

Our study is the first to describe the diverse patterns of LRR following comprehensive therapy in patients with TNBC according to different IHC-based molecular subtyping. Our results revealed a significantly high risk of LRR in the BLIS subtype (IHC-based BLIS; AR−, CD8−, FOXC1+) compared to the non-BLIS subtype. This study provides novel insights into the precision management of PMRT in different TNBC subtypes.

Previous studies reported that patients with TNBC had elevated LRR rates and shorter LRR times compared with patients with non-TNBC (19.2% vs 4.1%, 3.6 vs 5 months, respectively).^18^ Caudle et al^19^ indicated that the 5-year LRR for TNBC was higher than that of the Luminal A subtypes (10.5% vs 3%) in patients undergoing NAC, mastectomy, and PMRT. Richards et al^20^ estimated the 2-year LRR rate for patients with TNBC receiving PMRT to be 8%. Consistent with these findings, our study revealed an LRR of 14.9% in the BLIS group and 5.8% in the non-BLIS subtype. The results further delineate the characteristics of TNBC that are more likely to contribute to an increased risk of LRR.

In recent years, an increasing number of studies have focused on the relationship between biological subtypes and survival benefits following PMRT.^21^^,^^22^ Wang et al^23^ observed a significantly increased risk of LRR in patients with TNBC and HER2+, suggesting the value of biological subtype as a prognostic factor for LRR. The Keynote 522 study showed that neoadjuvant pembrolizumab combined with chemotherapy was beneficial for high-risk patients with TNBC^24^ and improved the prognosis of TNBC to a certain extent. Therefore, in-depth analysis of the biological heterogeneity of TNBC and expansion of clinical treatment options are of great clinical significance for reducing the risk of local-regional recurrence and improving efficacy. Our results refined the clinical characteristics of the BLIS subtype and indicated that the malignant phenotype of BLIS was associated with a higher rate of LR.

In the current study, the BLIS subtype was not statistically significantly associated with worse DFS compared with the non-BLIS subtype. Leeha et al^14^ showed that the BLIS subtype had a worse prognosis in patients with TNBC who underwent surgery and chemotherapy. The 5-year DFS and OS rates were 64.7% and 65.0%. This discrepancy could potentially be attributed to the utilization of PMRT in the present cohorts. A previous analysis reported a 5-year DFS of 97.1% for the entire population and that PMRT was associated with a favorable DFS.^25^

FOXC1 is a key biomarker for differentiating BLIS subtypes. It is a member of the FOX transcription factor family and plays an important role in cell growth, migration, invasion, and other biological processes.^26^ Studies have reported upregulated FOXC1 expression in basal-like breast cancer,^27^ with high levels of FOXC1 associated with poor prognosis in breast and other cancers.^28^^,^^29^ The underlying mechanism may be that FOXC1 is involved in tumor development and metastasis, including cell proliferation, epithelial-mesenchymal transition, and angiogenesis.^30^^,^^31^ The poor prognosis of the BLIS subtype may be related to the high expression of FOXC1. Further exploration is needed to ascertain the relationship between FOXC1 and the risk of LRR.

The 2013 St. Gallen International Breast Cancer Conference classified breast cancer into 4 subtypes based on immunohistochemical staining.^32^ In 2019, Jiang et al^11^ developed a new classification of breast cancer based on the genomic and transcriptomic profiling of 465 Chinese breast cancer cases. They designed the FUTURE trial (NCT03805399)^33^ to apply subtype-guided targeted therapy to refractory metastatic TNBC and found significant clinical benefits in those patients. With personalized cancer treatment emerging as a goal of oncology research worldwide, it is imperative to design individualized treatments according to tumor biological characteristics and risk categories.

Considering the heterogeneity of TNBC and the high LRR rate of the BLIS subtype, the consideration of risk-stratified RT intensification is warranted.^34^ For patients with BLIS, increasing the radiation dose or using radiosensitizing drugs can improve the efficacy of RT. Conversely, patients at lower risk of recurrence or those who achieve a pathologic complete response after NAC may benefit from RT de-escalation.

Our cohort of patients being treated before KEYNOTE-522 was published is a noteworthy limitation. Other limitations of our study include a small sample size. Because most patients were locally advanced, our results could not fully describe the whole clinical behavior of all TNBC stages. Inclusion of patients treated with breast-conserving surgery in future studies is warranted. Because of the nature of retrospective studies, selection bias may exist. Despite efforts to balance potential risk factors through multivariate analysis, the results may be affected by other unmeasured or unknown confounders.

## Conclusions

In conclusion, the present study demonstrated that the BLIS subtype had a higher risk of LRR than the non-BLIS subtype in patients with TNBC. IHC-based molecular subtyping holds promise as a prognostic factor to guide precision RT approaches in this population.

## Appendix Supplementary materials

Supplementary files(Unmarked) Image, application 1

## Footnotes

Supplementary material associated with this article can be found in the online version at doi:10.1016/j.adro.2025.101909.
