# Supplementary material for: Novel Molecular Subtyping Predicts Locoregional Recurrence in Triple-negative Breast Cancer
Source: Adv Radiat Oncol. 2025 Oct 2;10(12):101909. doi: 10.1016/j.adro.2025.101909 (PMC12666700; doi:10.1016/j.adro.2025.101909)
Supplement: Supplementary files(Unmarked) [file mmc1.docx]

**Table S1.**

**Univariate and multivariate Cox regression analysis of OS**

| Variables | | UVA (OS)  HR 95% CI | *P* value | MVA (OS)  HR 95% CI | *P* value |
| --- | --- | --- | --- | --- | --- |
| Age |  | 0.97 (0.94-1.00) | .05 | 0.98 (0.95-1.00) | .13 |
| Subtype | Non-BLIS | 1 | | 1 | |
|  | BLIS | 2.25 (1.18-4.32) | .01 | 2.16 (1.09-4.28) | .03 |
| Lymphvascular invasion | Positive | 1 | | 1 | |
|  | Negative | 0.58 (0.25-1.34) | .20 | 0.60 (0.24-1.46) | .25 |
|  | Unknown | 0.62 (0.19-2.05) | .43 | 0.50 (0.15-1.69) | .26 |
| Pathologic category, T | 0-2 | 1 | | 1 | |
|  | 3-4 | 3.41 (1.56-7.45) | < .01 | 2.93 (1.23-7.01) | .02 |
| Pathologic category, N | 1-2 | 1 | | 1 | |
|  | 3 | 5.03 (2.61-9.71) | < .01 | 4.87 (2.41-9.84) | < .01 |
| Grade | II | 1 | | 1 | |
|  | III | 0.71 (0.32-1.61) | .42 | 0.63 (0.27-1.49) | .30 |
|  | Unknown | 0.98 (0.37-2.62) | .97 | 1.16 (0.41-3.25) | .78 |

*Abbreviations: BLIS, basal-like immune-suppressed; CI, confidence interval; HR, hazard ratio; OS, overall survival.*

**Table S2.**

**Univariate and multivariate Cox regression analysis of DFS**

| Variables | | UVA (DFS)  HR 95% CI | *P* value | MVA (DFS)  HR 95% CI | *P* value |
| --- | --- | --- | --- | --- | --- |
| Age |  | 0.99 (0.97-1.01) | .34 | 1.00 (0.97-1.01) | .45 |
| Subtype | Non-BLIS | 1 | | 1 | |
|  | BLIS | 1.25 (0.78-2.02) | .36 | 1.24 (0.76-2.05) | .39 |
| Lymphvascular invasion | Positive | 1 | | 1 | |
|  | Negative | 0.58 (0.33-1.03) | .06 | 0.61 (0.33-1.13) | .12 |
|  | Unknown | 0.88 (0.43-1.77) | .71 | 0.77 (0.37-1.60) | .48 |
| Pathologic category, T | 0-2 | 1 | | 1 | |
|  | 3-4 | 2.29 (1.24-4.24) | < .01 | 2.17 (1.12-4.23) | .02 |
| Pathologic category, N | 1-2 | 1 | | 1 | |
|  | 3 | 4.01 (2.51-6.40) | < .001 | 3.56 (2.16-5.88) | < .001 |
| Grade | II | 1 | | 1 | |
|  | III | 0.82 (0.46-1.44) | .48 | 0.86 (0.47-1.56) | .86 |
|  | Unknown | 1.13 (0.58-2.22) | .70 | 1.54 (0.76-3.15) | .24 |

*Abbreviations: BLIS, basal-like immune-suppressed; CI, confidence interval; DFS, disease-free survival; HR, hazard ratio.*
